# The role of impaired vision and declined cognition in falls and fall-related risk factors among older people receiving home care in Finland—a cross-sectional register study

**DOI:** 10.1007/s10433-025-00860-2

**Published:** 2025-05-26

**Authors:** Tiina Pesonen, Heidi Siira, Visa Väisänen, Johanna Edgren, Satu Elo

**Affiliations:** 1 University of Oulu, Research Unit of Health Sciences and Technology, Aapistie 5, 90220 Oulu, Finland; 2 University of Oulu, Research Unit of Health Sciences and Technology/GeroNursing Centre, Oulu, Finland; 3 Finnish Institute for Health and Welfare, Helsinki, Finland; 4 Faculty of Social Sciences and Business Studies, Department of Health and Social Management, University of Eastern Finland, Kuopio, Finland; 5 Oulu University of Applied Sciences, University of Oulu, Oulu, Finland

**Keywords:** Home care, Vision impairment, Cognitive decline, Falls, Risk factors for falls

## Abstract

The risk of vision impairment and cognitive decline increases with age and can jeopardize safe ageing at home. Our aim was to investigate the association of impaired vision and cognitive decline with falls among older adults receiving home care. Additionally, we examined how previously identified fall-related risk factors are linked to vision impairment and cognitive decline. We used register data (n = 26 353) from the Finnish Resident Assessment Instrument (RAI) database. Four groups were created based on vision and cognition status: 1) no vision impairment or cognitive decline, 2) vision impairment alone, 3) cognitive decline alone, and 4) co-occurring vision impairment and cognitive decline. Multinomial logistic regression was used to examine the association of impaired vision and cognitive decline with falls. Based on results among older adults receiving home care, 12% had vision impairment alone, 37% had cognitive decline alone, and 18% had co-occurring vision impairment and cognitive decline. Vision impairment and cognitive decline, both individually and when co-occurring, were associated with experienced falls. Previously identified fall-related risk factors appeared to accumulate especially for older adults with co-occurring vision impairment and cognitive decline. Our study showed that vision impairment and cognitive decline play a significant role in the ability to live safely at home, particularly when they co-occur. Special attention should be paid to this vulnerable group to ensure safe and independent ageing in place. Timely identification and management of vision impairment may be crucial in preventing co-occurring vision impairment and cognitive decline.

**Supplementary Information:**

The online version contains supplementary material available at 10.1007/s10433-025-00860-2.

## Introduction

Vision impairment and cognitive decline are common health conditions among older people as ageing is a significant risk factor for both (Bai et al. 2022; Bourne et al. 2017; Livingston et al. 2024). These conditions can significantly impact daily living, leading to complex challenges in the ability to live independently (Jekel et al. 2015; Sloan et al. 2005) and increasing the need for home care services (Aljied et al. 2019; Burgdorf et al. 2022). Older people have a high prevalence of chronic eye conditions such as age-related macular degeneration (AMD), cataract, and glaucoma, of which conditions other than the dry form of AMD can be treated. Timely treatment can prevent permanent vision impairment. Furthermore, under/uncorrected refractive error, a treatable condition, is the leading cause of mild to moderate visual impairment worldwide (Burton et al. 2021). Based on a previous systematic review, the prevalence of vision impairment (including permanent and treatable) among home care clients ranged from 20 to 55% in a study conducted across 12 countries (Grue et al. 2010).

The prevalence of cognitive decline and dementia also increases exponentially with age, resulting in deterioration of comprehension, orientation, communication, and decision-making, i.e. *cognitive decline* (National Institutes of Health 2021). For example, the incidence of dementia, a progressive condition caused by neurodegenerative diseases, doubles every 5.8 years in high-income countries (Ali et al. 2015). In Finland, in 2021, 3% of individuals aged 65–74 were diagnosed with a memory disorder, whereas the prevalence increased to 41% among those aged 85 and older (Roitto et al. 2024). The systematic review showed that the prevalence of cognitive decline in community-dwelling older adults ranged between 5 and 41% (Pais et al. 2020). Comparison of the prevalence is complicated by the fact that the definition and assessment methods of vision impairment and cognitive decline vary between studies (Gauthier et al. 2006; WHO 2019).

The risk of falling also increases with age, and consequently, fall prevention has become an important issue in ageing societies (Montero-Odasso et al. 2022). Vision impairment and cognitive decline are identified as independent risk factors for falls (Chantanachai et al. 2021; Thomas et al. 2025) and may, therefore, threaten safe ageing in place. Furthermore, falls are the most common incidents among older people receiving home care (Tariq et al. 2015) and might have severe consequences for individuals, such as hip fractures, brain injuries, or even death (World Health Organization 2021). In addition to consequences for individuals, falls also result in significant economic costs on society (Heinrich et al. 2010). The risk factors for falls are multifaceted, as a wide range of demographic, physical, psychological, medical, socioeconomic, environmental, behavioural, and other aspects have been identified. The World Health Organization (2021) has introduced a risk factor model for falls in older age, encompassing four distinct aspects: 1) biological risk factors, such as older age, cognitive decline, female gender, and lower physical capacity; 2) socioeconomic risk factors, for example, lack of social interactions and low education level; 3) environmental risk factors, such as a hazardous home environment; and 4) behavioural risk factors, for example, multiple medication use. Fear of falling is also identified as a behavioural risk factor and might be a result of previous falls (Boelens et al. 2013). Living alone may hinder social interaction and can be a potential socioeconomic risk factor for falls, as living alone was associated with falls among older people in a previous systematic review (Petersen et al. 2020). Furthermore, a history of falling is identified as a strong predictor for future falls among older people (Montero-Odasso et al. 2021).

Falls are caused by a complex interaction of risk factors (World Health Organization 2008). In addition to impaired vision and declined cognition being an individual risk factor for falls among older people (Dhital et al. 2010; Muir et al. 2012), they are associated with many other risk factors for falls, such as older age, lower physical function (Lamoureux et al. 2004; Stavrinou et al. 2022), and lower education (Pais et al. 2020; Tolkkinen 2022). Additionally, fear of falling is associated with vision impairment (White et al. 2015). Furthermore, impaired vision and cognitive decline are bidirectionally associated with each other. Vision impairment has been associated with an increased risk of cognitive decline and dementia in older adults (Burton et al. 2021; Nagarajan et al. 2022; Shang et al. 2021). Conversely, cognitive impairment has been linked with greater vision impairment likelihood over time (Chen et al. 2021). However, there is limited information on these complex interactions of risk factors, and how co-occurring risk factors are associated with falls. When older individuals experience both vision impairment and cognitive decline, the challenges they face become more complex, and they might have increased difficulties with mobility or performing activities of daily living (Guthrie et al. 2018; Whitson et al. 2007). This may consequently raise the risk of falling and, as a result, hamper their safe ageing in place.

In Finland, as well as in other European countries, the care policy prioritizes home care as the primary option for older people in need of regular care and support (European Commission & Social Protection Committee, 2021; Ministry of Social Affairs and Health 2020). According to the Finnish national ageing policy, the aim is to enable older persons to live in their homes for as long as it is feasible from a safety perspective. Although research on the association between vision and cognition has increased in recent years, there is limited research specifically focusing on the co-occurrence of impaired vision and cognitive decline. Therefore, it is crucial to investigate how impaired vision and cognitive decline, both individually and co-occurring, are associated with falls among older adults receiving home care. Additionally, it is important to examine how previously identified fall-related risk factors are linked to vision impairment and cognitive decline to enhance understanding of the multifaceted connections between fall-related risk factors.

This study provides new evidence on a vulnerable group of home care clients with impaired vision and declined cognition, which, despite their considerable share of the home care population, have been understudied. Our results can help maintain the functioning of home care clients, enable safe ageing in place, and thus hinder the need for health and social care services. Furthermore, this research contributes to a better understanding of home care clients’ needs and can inform policymakers, healthcare providers, and caregivers about promoting safe ageing in place and improving the quality of care and life for this group.

## Methods

### Study design and population

This study employed a cross-sectional study design. The data were compiled from the National Resident Assessment Instrument (RAI) database, which is maintained by the Finnish Institute for Health and Welfare. The RAI system, developed by the international organization interRAI, consists of several standardized instruments to assess a person’s functioning and care needs (InterRAI 2023). Two RAI HC (Home care) instruments, RAI MDS-HC (Minimum Dataset) and interRAI HC, are specifically designed for use in home care and community settings.

In Finland, there were approximately 200,000 home care clients in 2021, with 72 per cent of them being over 75 years old (Finnish Institute for Health and Welfare 2023; Saukkonen et al. 2023). Home care services, which include both home nursing and home help, are provided by the well-being services counties, and majority of the personnel are practical nurses (74%), while 12 per cent are registered nurses (Finnish Institute for Health and Welfare 2023).

In 2021, the use of RAI instruments (mainly MDS-HC) in Finnish home care units was voluntary. While the units that did use RAI typically assessed all their clients, the national coverage was limited to 43 per cent for clients over the age of 75 due to the fact that not all units were using the instrument. However, starting from 1 April 2023, the use of the RAI instrument became mandatory for all Finnish home care and other long-term care service providers, as stated in the legislation (980/2012, 15 a §). Home care employees (usually practical nurses), together with clients and their close ones, carry out RAI assessments on average twice a year or more frequently if there are changes in the client’s condition. For this study, home care clients’ RAI data were extracted from the RAI database. The dataset included assessments of home care clients aged 65 or older that were conducted using the RAI MDS-HC instrument between 1 April and 30 September 2021 (one assessment period). The study population comprised 26,353 home care clients.

### Measures

*Vision impairment* was determined using a single RAI question, where vision is classified into 0–4 categories. Vision is assessed in sufficient lighting with current eyeglasses or other visual aids (e.g. magnifier), if used. Category 0 equals adequate vision, 1 indicates mild impairment, such as difficulty reading normal text in a book or newspaper, 2 represents moderate impairment, such as inability to see newspaper headlines but ability to see objects, 3 indicates highly impaired vision, where the person has difficulty identifying objects, but the eyes follow objects, and 4 means severe impairment including blindness, which appears so that the eyes do not follow objects (Morris et al. 2009). This study used vision impairment as a binary variable, with category 0 representing no vision impairment and categories 1–4 representing some level of vision impairment.

*Cognitive decline* was determined using the Cognitive Performance Scale (CPS, scale 0–6), which includes five items: comatose status, decision-making, short-term memory, making self-understood, and eating. In CPS, a score of 0 indicates intact cognition, 1 represents borderline intact cognition, 2 indicates mild impairment, 3 denotes moderate impairment, 4 corresponds to moderate/severe impairment, 5 signifies severe impairment, and 6 indicates very severe impairment, such as being in a comatose state (Morris et al. 1994). In this study, cognitive impairment was treated as a binary variable in the multinomial logistic regression analysis, with categories 0–1 representing no cognitive decline and categories 2–6 representing cognitive decline. The previous studies have utilized the same threshold (Onder et al. 2012; Yamada et al. 2016).

*Fall(s)* in the past 90 days was measured using a single question: *“Number of falls in the last 90 days (or since the last assessment if less than 90 days)”*. Falls were categorized into three groups to distinguish between different levels of fall frequency: The first group (0 falls) represented no falls, the second group (1–2 falls) represented occasional falls, and the third group (3 or more falls) represented multiple falls. In the 1–2 falls group, the mean number of falls was 1.3, and the median was 1, while in the 3 or more falls group, the mean was 4.8, and the median was 4, indicating significant differences between the groups.

In this study, *fall-related risk factors* were examined in four distinct aspects, as defined by the WHO. *Biological risk factors* measured were age, gender, and ADL (Activities of Daily Living) impairment. ADL impairment was determined using the ADL-H (Activities of Daily Living Hierarchy) scale, which measures functional ability in activities related to eating, personal hygiene, toilet use, and locomotion on a 7-point scale (0 for independent to 6 for total dependence). In this study, ADL impairment was used as a binary variable, with ADL-H categories 0–1 representing no impairment, and categories 2–6 representing ADL impairment (an individual has lost the ability to perform all of their ADLs independently). A similar definition has been used in previous research (Guthrie et al. 2018).

*Socioeconomic risk factors* in this study were low education and living alone. The original education level variable in the RAI included seven options: 1) no education, 2) primary school (former education), 3) comprehensive school, 4) vocational school, 5) high school, 6) university, and 7) no information on education. In this study, education levels were categorized into three groups: high education (vocational school or higher), low education (comprehensive school or lower), and no information on education. The “no information” category was included due to its high prevalence (26% of the sample). Living alone was determined using a single question including eight different options for living arrangements, for example, living with a spouse, children, parent, other relatives, or alone. Living alone was used as a binary variable.

*The environmental risk factor* measured was a hazardous home environment. A hazardous home environment was determined with a question which concerned things that make the home environment hazardous and unsafe for living. The question was used as a binary variable, yes meaning one or more of the following: 1) Lighting in the evenings (e.g. insufficient or missing lighting in the living room, bedroom, kitchen, toilet, or corridors; 2) floors and carpets (e.g. holes in the floor, electric cables in the walkways, or loose carpets; 3) bathroom and toilet (e.g. toilet out of order, leaking pipes, no necessary handrails, slippery bathtub, and outdoor toilet), and 4) kitchen (e.g. dangerous stove, broken refrigerator, rats, mice, or insects).

*The behavioural risk factor* measured was fear of falling, which was determined using an existing binary RAI variable (yes/no) based on the following question: “*Client limits going outdoors because he or she is afraid of falling”.*

### Statistical analysis

Descriptive statistics were conducted for all participant characteristics. Correlations between variables were explored to address the risk of multicollinearity. Four groups were created based on vision and cognition: 1) no vision impairment or cognitive decline, 2) vision impairment alone, 3) cognitive decline alone, and 4) co-occurring vision impairment and cognitive decline.

Links between previously identified fall-related risk factors, impaired vision, and cognitive decline were explored through a descriptive comparison of the prevalence of these risk factors across different vision and cognition status groups. In addition, the prevalence of these risk factors across different groups was compared to the average prevalence in the dataset. To determine whether the groups differ statistically in their fall-related risk factors, we used the chi-square test.

The association of impaired vision and declined cognition with falls was investigated using multivariate multinomial logistic regression, with falls (none, 1–2, and 3 or more in the past 90 days) as the outcome variable, and categorized vision and cognition status as the independent variables. The group of no falls was chosen as the reference group, which means that the odds ratios (ORs) presented should be interpreted in relation to the reference group. Previously identified fall-related risk factors, such as age, female gender, education level, ADL impairment, living alone, hazardous home environment, and fear of falling were entered into the model simultaneously. Missing values in living arrangements (n = 276) were handled using pair-wise deletion. The data were analysed using IBM SPSS version 28 for Windows. Statistical significance was defined at *p* < 0.05.

## Results

### Demographic data

Nearly half of the home care clients were over 85 years old, with a mean age of 83.3 years (SD 7.8), and a majority of them were female (68%) (Table 1). One-third of the home care clients had impaired vision, with most often mild impairment, while 8% had moderate or severe vision impairment (Supplementary material). Vision impairment, particularly moderate and severe impairment, increased with age. Among the younger age groups (65–74), vision impairment was more prevalent in men, while in the oldest age group (≥ 85), the prevalence was the same for both men and women.

**Table 1:** Characteristics of the home care clients

	Total n = 26 353	
	% (n)	Mean (SD)
Age		83.3 (7.8)
65–74	15.6 (4 098)	
75–84	35.8 (9 425)	
85 and older	48.7 (12 830)	
Gender		
Men	32.4 (8 542)	
Women	67.6 (17 811)	
Vision status (0–4)		0.4 (0.7)
Normal (0)	69.6 (18 334)	
Impaired (1–4)	30.4 (8 019)	1.4 (0.7)
Cognition status (0–6)		1.6 (1.3)
Normal (CPS 0–1)	44.9 (11 841)	0.4 (0.5)
Declined (CPS 2–6)	55.1 (14 512)	2.5 (0.9)
Activities of daily living (0–6)		0.9 (1.4)
Independent/minor supervision (0–1)	77.8 (20 493)	0.2 (0.4)
Impairment (2–6)	22.2 (5 860)	3.2 (1.0)
Education level		
Low education (comprehensive school or lower)	49.1 (12 941)	
High education (vocational school or higher)	24.5 (6 464)	
No information on education	26.4 (6 948)	
Living arrangement		
Living alone	77.3 (20 380)	
Living with someone	21.6 (5 697)	
NA	1.0 (276)	
Hazardous home environment		
No	84.3 (22 227)	
Yes	15.7 (4 126)	
Fear of falling		
No	57.8 (15 229)	
Yes	42.2 (11 124)	
Falls in the past 90 days		0.5 (1.2)
No	76.8 (20 238)	
1–2	18.0 (4 746)	1.3 (0.4)
3 or more	5.2 (1 369)	4.8 (2.3)

NA, not available; CPS, cognition performance scale; and SD, standard deviation

More than half of the home care clients had cognitive impairment, with 15% of them having moderate or severe cognitive impairment (Supplementary material). The prevalence of cognitive impairment increased with age, with a lower prevalence observed in the youngest age group (65–74). In all age groups, moderate to severe cognitive impairment was more common in men, while mild cognitive impairment was only more prevalent in men within the youngest age group.

The prevalence of ADL impairment was 22% among home care clients. Half of the home care clients had low education (comprehensive school or lower). It is worth noting that information on the education level was missing for every fourth client. Approximately one-fifth of the home care clients lived with someone, and one-sixth of the home care clients had a hazardous home environment. Over 40% of home care clients experienced a fear of falling. Nearly one-fourth had experienced a fall at least once in the past 90 days, while only 5% had fallen three times or more.

### Previously identified fall-related risk factors in different vision and cognition status groups

**Fig. 1:**
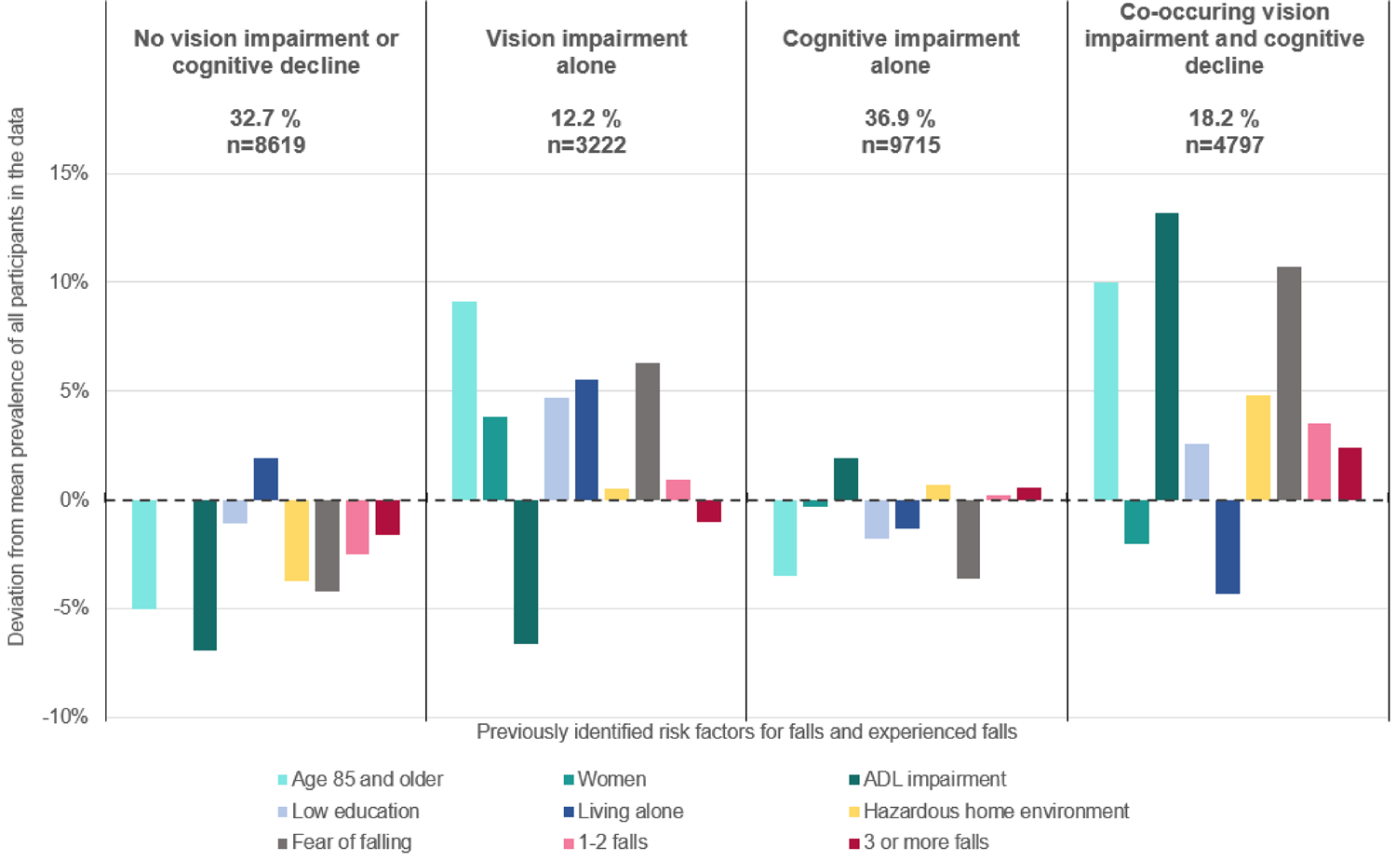
Comparison of the prevalence of fall-related risk factors and experienced falls across different vision impairment and cognitive decline groups, using the mean prevalence of all participants as the reference point

The links between previously identified fall-related risk factors, vision impairment, and cognitive decline were explored by comparing the prevalence of these risk factors across different vision and cognition status groups. The complete analysis is presented in the supplementary material. Figure 1 illustrates the deviations in risk factor prevalence and fall prevalence across different vision and cognition groups, compared to the overall mean prevalence among all participants.

Older adults with normal vision and cognition had lowest prevalence of all fall-related risk factors, except living alone. They also experienced fewer falls than persons with vision impairment and/or cognitive decline, as well as compared to the average older adult receiving home care. In contrast, older adults with vision impairment alone had a higher prevalence of many fall-related risk factors, such as older age, female gender, lower education, living alone, and fear of falling, compared to the average older adult receiving home care. However, they experienced approximately the same number of falls as the average home care client.

Among home care clients with cognitive decline alone, the prevalence of fall-related risk factors, except ADL impairment, and falls appeared to be at the same level as that of average home care client. Those with co-occurring vision impairment and cognitive decline had a considerably higher prevalence of older age, ADL impairment, hazardous home environments, and fear of falling compared to average home care clients. They also experienced more falls. However, they had a lower prevalence of female gender and living alone compared to the average home care clients.

### Association of vision impairment and cognitive decline with falls

The association of vision impairment and cognitive decline with falls was examined using multivariate multinomial logistic regression (Table 2). Vision impairment alone, cognitive decline alone, and co-occurring vision impairment and cognitive decline were categorized, using the group with no vision impairment or cognitive decline as the reference. The adjusted odds of 1–2 falls were 1.15 (95% CI: 1.03–1.28) times higher among clients with vision impairment alone, 1.21 (95% CI: 1.12–1.31) times higher among clients with cognitive decline alone, and 1.32 (95% CI: 1.20–1.45) times higher among clients with co-occurring vision impairment and cognitive decline. The adjusted odds of 3 or more falls were 1.16 (95% CI: 0.94–1.43) times higher among clients with vision impairment alone, although the result was not statistically significant, 1.55 (95% CI: 1.34–1.80) times higher among clients with cognitive decline alone, and nearly two times higher (OR:1.71 95% CI: 1.45–2.01) among clients with co-occurring vision impairment and cognitive decline. In addition, male gender, impaired ADL, living alone, a hazardous home environment, and fear of falling were associated with both 1–2 and 3 or more falls. Furthermore, low education increased the odds of experiencing three or more falls.

**Table 2:** The association between falls and vision impairment and cognitive decline among home care clients (n = 26 077) using multivariate multinomial logistic regression

(refr. no falls)	1–2 falls		3 or more falls	
	OR (95% CI)	OR (95% CI)	OR (95% CI)	OR (95% CI)
*Vision impairment or cognitive decline (ref. no vision impairment or cognitive decline)*				
Vision impairment alone	**1.15 (1.03–1.28)**	**0.013**	1.16 (0.94–1.43)	0.170
Cognitive decline alone	**1.21 (1.12–1.31)**	** < 0.001**	**1.55 (1.34–1.80)**	** < 0.001**
Co-occurring vision impairment and cognitive decline	**1.32 (1.20–1.45)**	** < 0.001**	**1.71 (1.45–2.01)**	** < 0.001**
Age	**1.01 (1.01–1.02)**	** < 0.001**	**0.99 (0.98–1.00)**	**0.025**
Sex (ref. male)	**0.93 (0.86–1.00)**	**0.039**	**0.72 (0.63–0.81)**	** < 0.001**
ADL impairment (ref. independent)	**1.31 (1.22–1.42)**	** < 0.001**	**2.64 (2.34–3.00)**	** < 0.001**
*Education level (ref. high education)*				
Low education	1.00 (0.92–1.09)	0.982	**1.18 (1.03–1.36)**	**0.016**
No information on education	0.97 (0.90–1.05)	0.455	1.10 (0.96–1.26)	0.168
Living alone (ref. living with someone)	**1.15 (1.06–1.25)**	** < 0.001**	**1.24 (1.08–1.42)**	**0.002**
Hazardous home environment (ref. no hazardous home environment)	**1.27 (1.17–1.38)**	** < 0.001**	**1.69 (1.48–1.93)**	** < 0.001**
Fear of falling (ref. no fear of falling)	**1.80 (1.68–1.92)**	** < 0.001**	**2.67 (2.37–3.01)**	** < 0.001**

OR, odds ratio; CI, confidence interval; and ADL, activities of daily living

Bolding indicates statistical significance at *p* < 0.05

## Discussion

The purpose of this research was to explore the association of vision impairment and cognitive decline with experienced falls among older adults receiving home care. Furthermore, we aimed to explore links between previously identified fall-related risk factors and vision impairment and cognitive decline. Previously identified risk factors were investigated from four different aspects, as outlined by the WHO (2021). The results suggest that previously identified fall-related risk factors tend to accumulate in home care clients with vision impairment alone, and especially in those with co-occurring vision impairment and cognitive decline. However, despite the accumulation of risk factors, the likelihood of falls was only slightly higher for those with vision impairment alone, whereas those with co-occurring vision impairment and cognitive decline had a significantly higher likelihood of experiencing falls.

Vision impairment was observed in 30% of home care clients with majority of the cases being mild. These results were consistent with a previous study conducted similarly within the home care setting using the RAI instrument (Grue et al. 2010). The prevalence of cognitive decline in this study was slightly higher compared to the findings of a previous systematic review (Pais et al. 2020). One explanation for the difference in prevalence could be the use of different assessment instruments or varying definitions and criteria for determining cognitive impairment. Furthermore, in our study, almost one-fifth of the home care clients had co-occurring vision impairment and cognitive decline. Comparing the prevalence to the previous studies is challenging due to a scarcity of research on co-occurring vision impairment and cognitive decline among home care clients. Our results support the findings of the previous studies that vision impairment and cognitive decline become more common with ageing (Bai et al. 2022; Bourne et al. 2017). Specifically, the prevalence of individuals aged 85 or older was notably higher in those with vision impairment alone or with co-occurring vision impairment and cognitive decline. This may suggest that co-occurring vision impairment and cognitive decline often progresses over time, indicating a bidirectional relationship between vision and cognition (Chen et al. 2021).

The results showed a hierarchical association of vision impairment and cognitive decline with falls. Compared to those with no vision impairment or cognitive decline, older adults with vision impairment alone had somewhat higher odds of experiencing falls, followed by those with cognitive decline alone, while those with co-occurring vision impairment and cognitive decline had the highest odds of experiencing falls. In addition, living alone, ADL impairment, fear of falling, and hazardous home environment increased the odds of experiencing falls, supporting the findings of the previous studies (Boelens et al. 2013; World Health Organization 2008). In addition, low education was associated with 3 or more falls in this study. Contrary to the previous studies, our results revealed males had increased odds of falls (Montero-Odasso et al. 2022; World Health Organization 2008). Age, on the other hand, was inconsistently associated with falls, and the changes in odds values were minor. This may reflect the relatively high mean age in this study data, as included home care clients were at least 65 years old.

It has been previously established that as exposure to fall risk factors increases, the risk of falling and injuries becomes greater (World Health Organization 2008). However, our study demonstrated that although the prevalence of several risk factors was higher in the group with vision impairment alone, the odds of falls were lower compared to those in the group with cognitive decline alone. For example, the prevalence of living alone, which was associated with increased odds of experiencing falls, particularly 3 or more falls, was highest among older adults with vision impairment alone, but lower than average in those with cognitive decline alone. The high prevalence of living alone among those with vision impairment alone may be explained by the fact that individuals with impaired vision who live alone are more likely to require assistance from home care services to be able to continue living at home. In other words, a person with impaired vision alone who lives with someone is less likely to need a home care service than the one who lives alone.

Based on the results, ADL impairment strongly increased the odds of falls, particularly 3 or more falls. Prevalence of ADL impairment varied between vision and cognitive status groups. Prevalence was lower than average in those with either normal vision and cognition or vision impairment alone, slightly higher in those with cognition decline alone, and notably higher with co-occurring vision impairment and cognition decline. The previous studies support this finding, showing that visually impaired people need help, especially with instrumental activities of daily living, such as taking medication (Weeraratne et al. 2012), rather than with activities of daily living (Guo et al. 2021). Additionally, older adults with cognitive decline alone, or with co-occurring vision impairment and cognitive decline, have been found to have a higher prevalence of ADL impairment compared to those with vision impairment alone (Guthrie et al. 2018), increasing the need for care (Burrell et al. 2023). As the need for care increases, it may result in a situation where living at home would not be possible without living with someone. This may explain why the prevalence of living alone was lower among those with co-occurring vision impairment and cognitive decline compared to the average.

The prevalence of hazardous home environment, which was associated with falls, was notably higher among older adults with co-occurring vision impairment and cognitive decline. Since both impaired vision and cognitive decline hinder environmental perception (Javaid et al. 2016; Lord and Dayhew 2001), their co-occurrence could reduce older adults’ ability to maintain a safe home environment. As a result, targeted interventions might be necessary, which could include improving accessibility, lighting, and contrasts at home (Barstow et al. 2011), as well as formal or informal support—especially for those living alone. On the other hand, we should not forget those who live with someone, as in most cases, the spouse or spousal caregiver might have their own disabilities or care needs, which can impact the available resources and support. Therefore, recognizing the resources of the spouse or spousal caregiver is crucial for providing holistic care to older adults with co-occurring vision impairment and cognitive decline, while ensuring everyone’s well-being.

The prevalence of fear of falling, which was strongly associated with falls, was notably high among older adults with vision impairment alone. Similar results have been found in a previous study, which suggests that fear of falling is more prevalent among visually impaired older adults than their normally sighted peers (White et al. 2015). The prevalence of fear of falling was also high among clients with co-occurring vision impairment and cognitive decline. Increased fear may lead to reduced mobility, thereby affecting physical and social functioning (Cumming et al. 2000; Deshpande et al. 2008). This, in turn, may result in greater ADL impairment and a higher risk of falling, revealing the multifaceted connections between fall-related risk factors and falls. Fear can also contribute to an increased mental load and can affect the quality of life, social well-being, and participation in the living environment and society. Home care clients with vision impairment who also experience a fear of falling should be encouraged and supported to engage in versatile mobility and balance training, learn new compensatory skills to move safely despite their disability, and possibly start using mobility aids, such as a white cane. In addition, correcting impaired vision, for example, with glasses, when possible, may help prevent falls and thereby reduce the fear of falling, as a history of falls is strongly associated with the fear of falling (Boelens et al. 2013).

The care policy for older people across Europe emphasizes ageing in place. Therefore, it is paramount to investigate the factors that enable safe living at home. Our findings suggest that co-occurring vision impairment and cognitive decline is a combination in which not only do fall risk factors accumulate, but the likelihood of experiencing falls also increases. Therefore, it is important to consider co-occurring vision impairment and cognitive decline when assessing fall risk or creating an individual care plan for a home care client, as this significantly impacts their ability to live safely at home. Additionally, efforts should be made to prevent the development of co-occurring vision impairment and cognitive decline. One key process is the identification, treatment, and rehabilitation of vision impairment. Interventions aimed at preventing vision impairment are also essential as it has been identified as a risk factor for dementia (Livingston et al. 2024). By preventing vision impairment, it may also be possible to prevent or slow down cognitive decline. Based on our findings, we recommend including vision assessments in both current and future fall prevention programmes.

Preventing vision impairment is particularly important through timely identification and proper care for eye diseases that can lead to severe vision impairment. Based on previous research, vision impairment can be easily identified in the home environment through a simple screening test (van Nispen et al. 2019). In addition, vision impairment may be due to under- or uncorrected refractive error (Burton et al. 2021). According to a previous study, up to half of the vision impairments in individuals with memory disorders living in community and care homes could be corrected with up-to-date eyeglasses (Bowen et al. 2016). In addition, when vision and cognitive impairments cannot be prevented, their negative effects on safe living can be reduced by paying attention to the home environment, ensuring accessibility, proper lighting, sufficient contrasts, and cleanliness. Collaboration between home care and eye health care professionals needs to be increased to ensure the timely identification of impaired vision and the provision of low vision rehabilitation interventions for those who are entitled to and in need of them, along with providing additional training for home care employees on the importance of vision for the safe living of older adults.

## Strength and limitations

One of the key strengths of this study lies in its comprehensive and representative dataset, as well as its unique register-based approach in nursing science research. In addition, the novelty of our investigation is a notable strength, as it focuses on the relatively unexplored area of co-occurring vision impairment and cognitive decline. Furthermore, RAI instruments are standardized and have been proven reliable and valid through rigorous research and testing (Hirdes et al. 2008; Landi et al. 2000). However, the assessment produced by the RAI is subjective and vision and hearing, for example, are not evaluated using objective measures, such as a visual acuity test or audiometry. In addition, we were unable to verify the competence of the assessors in performing RAI assessments. Another limitation to consider is that the characteristics of the home care receivers in Finland may differ from other countries, in which case the results are not necessarily applicable on a global scale. Additionally, the data do not include all home care clients in Finland since only those with RAI assessments were included. Furthermore, the used variables might not comprehensively encompass all the risk factors for falling. For instance, various medications can influence falls. The study also did not consider impaired hearing, which, together with impaired vision, is known to increase challenges for independent living at home. Next, using alternative cut-off values, such as different categories for falls, could have yielded different results. Consequently, we performed a sensitivity analysis using 1 fall as the threshold for occasional falls and 2 or more for multiple falls. The results were similar in terms of effect sizes and directions. Lastly, the cross-sectional design of this study restricts our ability to make causal interpretations of the findings.

## Conclusion

This study revealed that vision impairment and cognitive decline increase the likelihood of falls, especially when co-occurring, compromising the ability to live safely at home. Fall-related risk factors also appear to accumulate in older adults with co-occurring vision impairment and cognitive decline, potentially leading to a higher number of falls. This vulnerable subpopulation of home care clients deserves special attention to ensure their safe ageing in place. Timely identification and management of vision impairment are paramount, as they can prevent the co-occurring vision impairment and cognitive decline, thereby reducing the risk of falls. Finally, co-occurring vision impairment and cognitive decline should be considered when assessing fall risk or planning individual care for a home care client to ensure adequate care and promote safe ageing in place.

## Supplementary Information

Below is the link to the electronic supplementary material.

Supplementary file1.

## Data Availability

The dataset generated during the current study is not publicly available. However, the dataset is available from the corresponding author on reasonable request.
