# Supplementary material for: The role of impaired vision and declined cognition in falls and fall-related risk factors among older people receiving home care in Finland—a cross-sectional register study
Source: Eur J Ageing. 2025 May 26;22(1):22. doi: 10.1007/s10433-025-00860-2 (PMC12106257; doi:10.1007/s10433-025-00860-2)
Supplement: Supplementary file 1 — Supplementary file1. [file 10433_2025_860_MOESM1_ESM.docx]

**The role of impaired vision and declined cognition in falls and fall-related risk factors among older people receiving home care in Finland** **- a cross-sectional register study**

Pesonen, T.*^1^, Siira, H. ^1^, Väisänen, V. ^2,3^, Edgren, J. ^2^ & Elo, S. ^1,4^

^1^ University of Oulu, Research Unit of Health Sciences and Technology, ^2^ Finnish Institute for Health and Welfare, Helsinki, Finland, ^3^ University of Eastern Finland, Faculty of Social Sciences and Business Studies, Department of Health and Social Management, and ^4^ Oulu University of Applied Sciences. *Corresponding author

**Supplementary Information**

| 1. Prevalence of vision impairment and cognitive decline in different age groups. | | | | | | |  |
| --- | --- | --- | --- | --- | --- | --- | --- |
|  | **Age groups** | | | | | |  |
|  | **65-74** | | **75-84** | | **85 and older** | | **TOTAL** |
|  | **Male** n=2029 | **Female** n=2069 | **Male** n=3310 | **Female** n=6115 | **Male** n=3203 | **Female** n=9627 | n=26 353 |
| Vision, % (n) |  |  |  |  |  |  |  |
| No impairment (0) | 75.6 (1533) | 79.0 (1634) | 72.4 (2397) | 75.5 (4619) | 63.7 (2041) | 63.5 (6110) | 69.6 (18334) |
| Mild (1) | 20.0  (405) | 17.3 (357) | 21.3  (706) | 19.6 (1201) | 26.9  (861) | 25.9 (2489) | 22.8 (6019) |
| Moderate/severe (2-4) | 4.5  (91) | 3.8  (78) | 6.3  (207) | 4.8  (295) | 9.4  (301) | 10.7 (1028) | 7.6 (2000) |
| p-value* | **p = 0.032** | | **p < 0.001** | | p = 0.091 | |  |
| Cognition, % (n) |  |  |  |  |  |  |  |
| No decline (CPS 0-1) | 48.4  (983) | 54.6 (1130) | 42.2 (1397) | 44.3 (2706) | 41.6 (1334) | 44.6 (4291) | 44.9 (11841) |
| Mild (CPS 2) | 36.4  (738) | 33.1 (684) | 39.8 (1319) | 40.7 (2489) | 40.5 (1297) | 41.1 (3959) | 39.8 (10486) |
| Moderate/severe (CPS 3-6) | 15.2  (308) | 12.3 (255) | 17.9  (594) | 15.0 (920) | 17.9  (572) | 14.3 (1377) | 15.3 (4026) |
| p-value* | **p < 0.001** | | **p = 0.001** | | **p < 0.001** | |  |
| *Chi-square test | | | | | | | |

**2. Prevalence of falls and biological, socioeconomic, environmental, and behavioural fall-related risk factors in different vision and cognition status groups.**

|  | No impairment  32.7 %  n=8619 | Vision  impairment alone  12.2 %  n=3222 | Cognitive decline alone  36.9 %  n=9715 | Co-occurring vision impairment and cognitive decline  18.2 %  n=4797 |  |
| --- | --- | --- | --- | --- | --- |
|  | **% (n)** | | | | **p-value*** |
| Falls in the last 90 days |  |  |  |  | <0.001 |
| No | 80.9 (6976) | 76.9 (2478) | 76.0 (8126) | 70.9 (3403) |  |
| 1-2 | 15.5 (1337) | 18.9 (608) | 18.2 (1770) | 21.5 (1031) |  |
| 3 or more | 3.6 (306) | 4.2 (136) | 5.8 (564) | 7.6 (363) |  |
| Biological risk factors |  | | | |  |
| Age (years) |  |  |  |  | <0.001 |
| 65-74 | 19.7 (1695) | 13 (418) | 15.2 (1472) | 10.7 (513) |  |
| 75-84 | 36.7 (3161) | 29.2 (942) | 39.7 (3855) | 30.6 (1467) |  |
| ≥ 85 | 43.7 (3763) | 57.8 (1862) | 45.2 (4388) | 58.7 (2817) |  |
| Gender |  |  |  |  | <0.001 |
| Men | 32.4 (2794) | 28.6 (920) | 32.7 (3177) | 34.4 (1651) |  |
| Women | 67.6 (5825) | 71.4 (2302) | 67.3 (6538) | 65.6 (3146) |  |
| Activities of Daily Living |  |  |  |  | <0.001 |
| Independent/minor supervision (0-1) | 84.7 (7300) | 84.4 (2719) | 75.9 (7373) | 64.6 (3101) |  |
| Impairment (2-6) | 15.3 (1319) | 15.6 (503) | 24.1 (2342) | 35.4 (1696) |  |
| Socioeconomic risk factors |  |  |  |  |  |
| Education, % (n) |  |  |  |  |  |
| Low | 48.0 (4135) | 53.8 (1735) | 47.3 (4591) | 51.7 (2480) | <0.001 |
| High | 25.7 (2216) | 22.2 (714) | 25.6 (2486) | 21.8 (1048) |  |
| No information | 26.3 (2268) | 24.0 (773) | 27.2 (2638) | 26.5 (1269) |  |
| Living arrangement, % (n) |  |  |  |  | <0.001 |
| Living alone | 79.2 (6829) | 82.8 (2668) | 76.0 (7379) | 73.0 (3504) |  |
| Living with someone | 19.5 (1680) | 16.2 (522) | 23.1 (2251) | 26.0 (1244) |  |
| NA | 1.3 (110) | 1.0 (32) | 0.9 (85) | 1.0 (49) |  |
| Environmental risk factor |  |  |  |  |  |
| Hazardous home environment, % (n) |  |  |  |  | <0.001 |
| No | 88.0 (7587) | 83.8 (2699) | 83.6 (8126) | 79.5 (3815) |  |
| Yes | 12.0 (1032) | 16.2 (523) | 16.4 (1589) | 20.5 (982) |  |
| Behavioural risk factor |  |  |  |  |  |
| Fear of falling, % (n) |  |  |  |  | <0.001 |
| No | 62.0 (5344) | 51.5 (1658) | 61.4 (5968) | 47.1 (2259) |  |
| Yes | 38.0 (3275) | 48.5 (1564) | 38.6 (3747) | 52.9 (2538) |  |
| NA= Not available  *Chi-square test |  |  |  |  |  |
